# Maternal serum per- and polyfluoroalkyl substances during pregnancy and breastfeeding duration: Erratum

**DOI:** 10.1097/EE9.0000000000000276

**Published:** 2023-10-26

In the article in the June 2023 compendium, the authors identified an error with the reporting of PFAS concentrations below the limit of detection (LOD). For concentrations reported by the laboratory as <LOD, they used instrument values when available. If values were unavailable or reported as zero, they were replaced by the LOD/2. For some values of PFDA reported as 0.1 ng/mL, the authors incorrectly described these values as detectable although the laboratory had labeled them as <LOD. This error does not affect the main analytic results or findings of the paper.

However, the following are corrections to the text:

1. The Methods section should have stated, “For this analysis, we included only PFAS detectable in >60% of participants, which included PFHxS, PFOS, PFOA, PFNA, and PFDA.”
2. In Table 2, the first quartile (Q1) of PFDA should have been reported as “<LOD.”
